# The Chaperone TRAP1 As a Modulator of the Mitochondrial Adaptations in Cancer Cells

**DOI:** 10.3389/fonc.2017.00058

**Published:** 2017-03-29

**Authors:** Ionica Masgras, Carlos Sanchez-Martin, Giorgio Colombo, Andrea Rasola

**Affiliations:** ^1^Dipartimento di Scienze Biomediche, Istituto di Neuroscienze, Consiglio Nazionale delle Ricerche (CNR), Università di Padova, Padova, Italy; ^2^Istituto di Chimica del Riconoscimento Molecolare, Consiglio Nazionale delle Ricerche (CNR), Milano, Italy

**Keywords:** tumor necrosis factor receptor-associated protein 1, tumor metabolism, heat shock proteins, mitochondria, reactive oxygen species, post-translational modifications, kinase, allosteric ligands

## Abstract

Mitochondria can receive, integrate, and transmit a variety of signals to shape many biochemical activities of the cell. In the process of tumor onset and growth, mitochondria contribute to the capability of cells of escaping death insults, handling changes in ROS levels, rewiring metabolism, and reprograming gene expression. Therefore, mitochondria can tune the bioenergetic and anabolic needs of neoplastic cells in a rapid and flexible way, and these adaptations are required for cell survival and proliferation in the fluctuating environment of a rapidly growing tumor mass. The molecular bases of pro-neoplastic mitochondrial adaptations are complex and only partially understood. Recently, the mitochondrial molecular chaperone TRAP1 (tumor necrosis factor receptor associated protein 1) was identified as a key regulator of mitochondrial bioenergetics in tumor cells, with a profound impact on neoplastic growth. In this review, we analyze these findings and discuss the possibility that targeting TRAP1 constitutes a new antitumor approach.

## Mitochondrial Metabolism in Cancer

**Figure 1:**
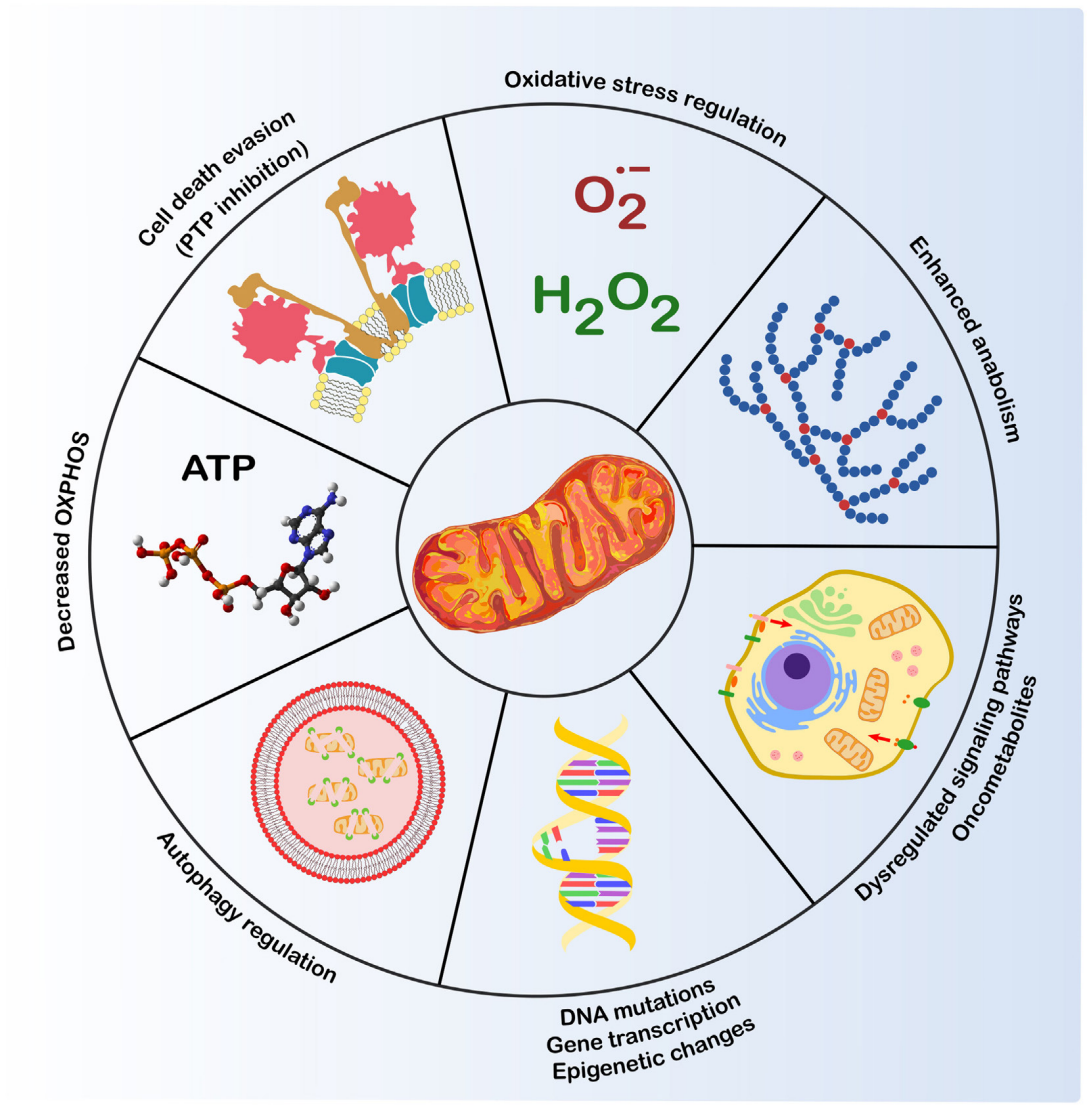
**Mitochondria and cancer**. Mitochondria play a crucial role in several biological routines involved in tumorigenesis (1): control of ROS levels, whose increase can lead to DNA mutations and genomic instability; autophagy regulation; resistance to cell death stimuli; metabolic changes, such as decreased oxidative phosphorylation (OXPHOS) and induction of anabolic pathways; dysregulation of transduction pathways, including kinase signaling and changes in oncometabolite levels that modulate transcription factors and epigenetic changes.

Mitochondria take part actively in the process of neoplastic transformation as critical components in the regulation of cell survival, redox equilibrium, autophagy, and core metabolic pathways (Figure 1) (1). Changes in each of these biological routines are required for cell progression to malignancy, allowing to escape death signals, to cope with oxidative stress, and to meet energy demands for growth and proliferation in an environment where nutrient and oxygen can be spatially and temporally heterogeneous (2–4). The profound metabolic rewiring of tumor cells is driven by alterations in multiple signaling pathways that deeply intertwine mitochondrial changes with rearrangements occurring in other cellular compartments. These pathways are mastered by activation of oncogenes such as Ras or Myc (5, 6), induction of transcription factors such as HIF1α (7), and inactivation of tumor suppressor genes such as p53 (8, 9). In most tumor cell types, this leads to enhanced glucose utilization paralleled by inhibition of oxidative phosphorylation (OXPHOS) irrespective of oxygen availability, a rearrangement also known as aerobic glycolysis or Warburg effect (10–12). Induction of pathways that branch from glycolysis, including the pentose phosphate pathway (13) or amino acid biosynthetic pathways (14, 15) also affords high levels of anabolic intermediates, whereas the increased activity of ROS scavenging systems shields tumor cells from potentially lethal oxidative insults (16, 17), and acidification of the extracellular microenvironment increases the activity of several pro-invasive factors (18, 19). To fulfill their anabolic needs, tumor cells also stimulate lipid biosynthesis (20) and increase the usage of glutamine as an anaplerotic mechanism to fuel the tricarboxylic acid (TCA) cycle and to provide nitrogen and carbon (21). As a result, the neoplastic cell proliferates even in the core of an expanding tumor mass, where it can face shortages of blood supply leading to hypoxia, paucity of nutrients, and oscillations of redox conditions (2, 10, 22).

In this complex scenario, mitochondria are not only the effector devices of the bioenergetic adaptations of tumor cells, but also active inducers of their metabolic rewiring. For instance, inactivating mutations in genes encoding respiratory complex subunits have been associated with a wide range of cancer types (23–25). However, the neoplastic role played by these mutations is unclear, as most cancer cells are able to generate ATP through a competent OXPHOS (26). Thus, more subtle molecular mechanisms might be responsible for mitochondrial changes in cancer cells. One example is provided by the increase in intracellular levels of oncometabolites, i.e., metabolic intermediates whose accumulation can drive pro-neoplastic programs (27, 28). Loss-of-function mutations in the TCA cycle enzymes succinate dehydrogenase (SDH) or fumarate hydratase, cause the accumulation of succinate or fumarate, respectively, leading to the onset of a specific subset of tumors, including paraganglioma, pheochromocytoma and renal cell carcinoma (29). In addition, gain-of-function mutations in isocitrate dehydrogenase (IDH) cause the accumulation of 2-hydroxyglutarate, which plays an important role in glioma, acute myeloid leukemia, chondrosarcoma and cholangiocarcinoma (28). The pro-neoplastic activity of these oncometabolites is complex and probably not entirely understood yet (27), spanning from induction of transcription factors such as HIF1α (25, 29) to epigenetic modifications (30) and miRNA regulation leading to epithelial-to-mesenchymal transition and invasiveness (31).

In addition to the conceptual interest and to the mechanistic insights provided by the work on oncometabolite functions, these observations highlight the importance of mitochondria-driven tuning of metabolic features in neoplastic cells. It is conceivable to envision that mitochondrial metabolic rearrangements are complex and highly dynamic process on the route to tumor onset and growth, which might require fast and flexible adaptations to environmental changes well beyond mutations targeting components of the bioenergetic circuitries. Therefore, components of the mitochondrial metabolic machinery might be rapidly regulated during tumorigenesis, for instance, by post-transcriptional modifications such as phosphorylation events or chaperone-mediated tuning of their activity. In this review, we analyze how one of these components, the mitochondrial chaperone TRAP1, contributes to the neoplastic process and how TRAP1 targeting might be a new promising therapeutic approach for cancer treatment.

## The Molecular Chaperone TRAP1

In the last few years, several observations have pointed toward an important role played by TRAP1 in the adaptive metabolic changes of tumor cell mitochondria. TRAP1 was first identified as an interactor of the type 1 tumor necrosis factor receptor-1 (32) and later as heat shock protein 75 (HSP75) (33). It is a molecular chaperone of the heat shock protein 90 (HSP90) family, as TRAP1 and HSP90 display 34% identity and 60% homology at the mRNA level and share the same domain organization (34). The TRAP1 cognate HSP90 is crucial in maturation, activation, and stabilization of a large set of proteins, including components of cell cycle, apoptosis, and motility programs, whose deregulation is crucial in tumorigenesis (35, 36). Therefore, HSP90 has emerged as a promising target for development of anticancer drugs (37). In more recent years, the observation that TRAP1 expression is induced in several tumor types (38), together with its similarity with HSP90, have sparked interest in better understanding the mode of action and the potential role(s) in the neoplastic process played by this mitochondrial chaperone.

### A TRAP(1) for Keeping Clients in Shape

TRAP1 expression seems to be mainly restricted to mitochondria (1). Indeed, TRAP1 is the only HSP90-family member that contains a 59-amino acid, N-terminal mitochondrial import sequence that is removed upon organelle import (34). TRAP1 acts as a homodimer that utilizes ATP to carry out its chaperone activity. Each of the two protomers is formed by three major domains: the N-terminal domain, responsible for ATP binding and hydrolysis; the C-terminal domain (CTD) that provides a dimerization interface between protomers; and the middle domain (M-domain) that completes the ATP-pocket and contains the recognition surface for the chaperone substrates, called clients. Thus, the M-domain directly couples ATP binding and hydrolysis with client remodeling (39). Unlike other eukaryotic HSP90 paralogs, TRAP1 lacks the charged linker between the middle and C-terminal domains and features a long extension of the N-terminal β-strand (the so-called “strap”) that crosses between protomers in the closed state and acts as a thermal regulator of protein function, inhibiting it at low temperatures. However, the most distinctive trait of TRAP1 is the presence of a marked asymmetric conformation, as one protomer is reconfigured *via* a helix swap at the middle:C-terminal domain (M-domain:CTD) interface (40).

**Figure 2:**
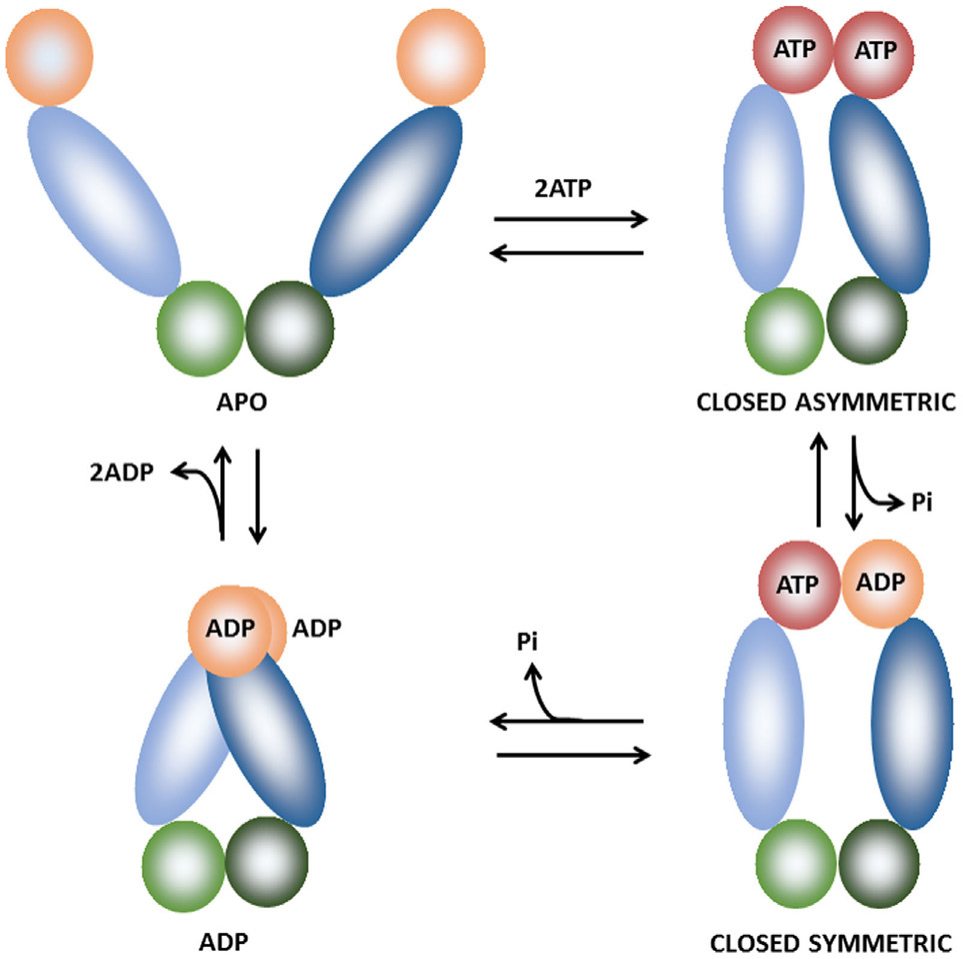
**Schematic representation of the of the TRAP1 conformational cycle**. TRAP1 protomers are shown in different hues and colored orange [ADP-bound or naked N-terminal domain (NTD)], red (ATP-bound NTD), blue [middle domain (MD)], and green [C-terminal domain (CTD)]. In the absence of bound nucleotides (apo state), TRAP1 populates a number of states with open conformations. Upon ATP binding, the chaperone shifts to an asymmetric closed conformation with significant strain, leading to buckling of the MD:CTD interface. After hydrolysis of the first ATP, strain is relieved and the MD:CTD interface is rearranged, forming a symmetric state. Hydrolysis of the second ATP leads to the formation of the ADP state. The cycle eventually returns to the open conformation after ADP release (39–42).

The ATPase cycle of TRAP1 has been investigated in detail (39, 41, 42) allowing to establish a model for its conformational cycle. Both TRAP1 protomers undergo concerted structural changes through rounds of ATP binding, hydrolysis, and release (39, 42), although it is unknown how ATP hydrolysis is coupled to client maturation. During the ATPase cycle, TRAP1 can adopt three distinct states (Figure 2): an open conformation, called the *apo* state; a closed conformation with an N-terminal strap/extension straddled between both protomers and a coiled-coil, intermediate conformation with the N-terminal domains in close physical proximity (40, 42). ATP binding induces a dramatic structural change in the chaperone configuration leading to the formation of a closed asymmetric conformation (39, 43). The subsequent ATP hydrolysis gives off the energy required for client remodeling and is performed in a two-step process (39). The hydrolysis of the first ATP causes changes in protomer symmetry that lead to the rearrangement of the client-binding site, which in turn is coupled to structural changes in the client conformation. The second ATP is used to induce the formation of a compact ADP state of the chaperone, which releases the client and eventually the ADP molecules. So far the number of known clients of TRAP1 is quite small (38) and TRAP1 co-chaperones, i.e., proteins that assist and regulate the chaperone cycle, have yet to be identified.

### TRAP1 Functions: Mitochondrial Homeostasis

A detailed analysis of the tissue expression profile of TRAP1 is lacking and little is known on its physiological functions. Early experiments have shown that TRAP1 displays an anti-oxidant activity. Indeed, reduced TRAP1 expression corresponds to increased ROS levels and enhanced susceptibility to cell death upon oxidative stress, whereas exposure to sublethal doses of oxidants increases TRAP1 protein levels (44–47). This involvement of TRAP1 in mitochondrial redox control has been linked to the pathogenesis of several disorders, among which there are the Parkinson’s disease (PD) and ischemic damage. In PD, redox unbalance and mitochondrial dysfunction play key roles (48). TRAP1 could protect mitochondria by reversing cytotoxicity mediated either by α-synuclein, whose accumulation is pathogenic in PD (49) or by alterations in the Parkin/PINK1 axis, which controls mitochondrial homeostasis (50, 51). In ischemia, the antioxidant activity of TRAP1 could decrease mitochondrial dysfunction, thus reducing damage both in brain (52) and in heart (53, 54). These observations, together with reports of a TRAP1 involvement in the regulation of mitochondrial dynamics (55) and mitophagy (50, 51) have led to the hypothesis that TRAP1 would have an important role in maintenance of mitochondrial homeostasis under specific stress or pathological conditions, including those encountered by cells during the deregulated growth of a tumor mass.

In spite of these protective functions exerted by TRAP1, its overexpression in transgenic mice leads to fatty liver and increased inflammation after partial hepatectomy (PH) (56). Indeed, PH is a stress factor that boosts proliferation and profound metabolic changes in hepatocytes, which is somehow reminiscent of neoplastic growth, but expression levels of TRAP1 do not change after PH in wild-type animals (57). Therefore, the importance of TRAP1 in responding to stress conditions and its modes of regulation are probably context dependent, a concept that could be crucial for understanding TRAP1 functions in tumorigenesis.

## A Metabolic TRAP in cancer

TRAP1 expression is higher in many tumors compared to surrounding non-malignant tissues (1, 38). Elevation of TRAP1 protein levels correlates with malignant progression and metastasis in several neoplastic models, including prostate and breast cancer, hepatocellular carcinoma (HCC), and colorectal carcinoma (57–61), and with disease recurrence in non-small cell lung cancer (62). Hence, in these malignancies, TRAP1 is a candidate biomarker for cancer progression and for prognosis outcome. These studies open the possibility that TRAP1 activity could in some way favor neoplastic growth, in line with observations that levels of several chaperones increase in a wide range of cancer types, where they contribute to uncontrolled growth, enhanced survival, and acquisition of angiogenic and metastatic potential (63). Understanding whether TRAP1 has any tumorigenic role could help in elucidating how mitochondria can contribute to the neoplastic process and could possibly pave the way for novel antitumor strategies.

### TRAP1 and the Warburg Phenotype: For Many but Not for All

**Figure 3:**
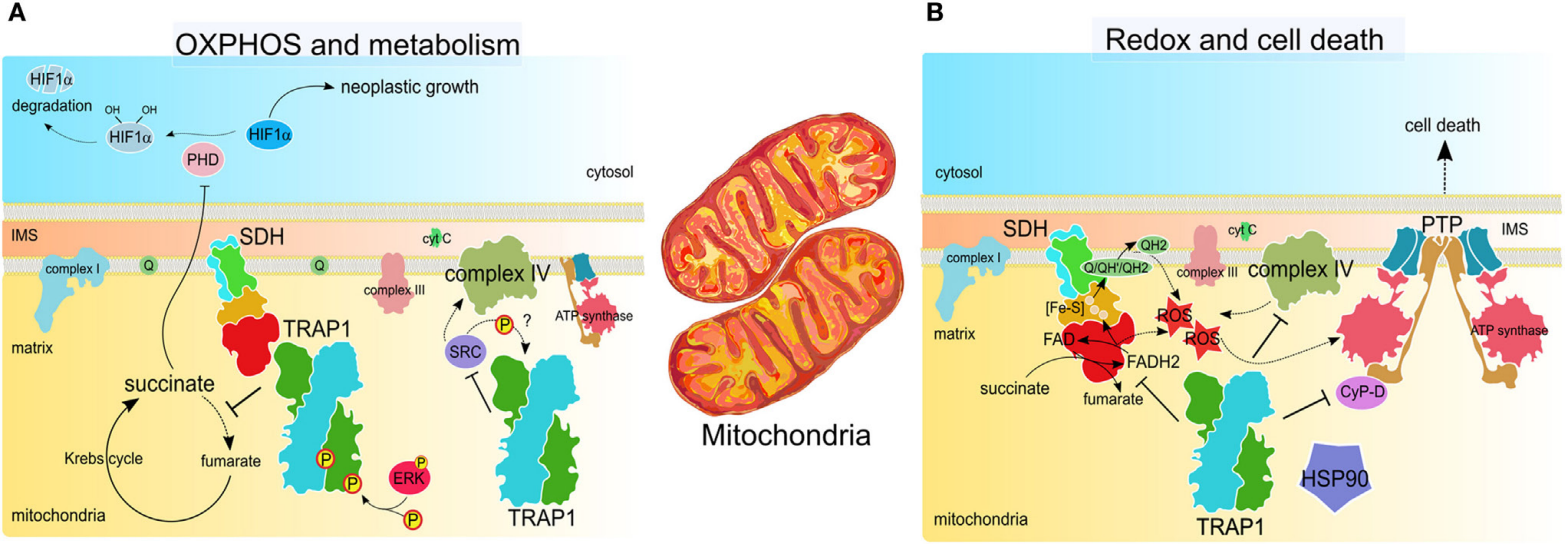
**TRAP1 activity in tumor cell mitochondria**. **(A)** TRAP1 inhibits oxidative phosphorylation (OXPHOS) by interacting with both succinate dehydrogenase (SDH) and cytochrome oxidase, *aka* complex II and complex IV of the respiratory chain, respectively. SDH inhibition is enhanced by ERK1/2-dependent phosphorylation of TRAP1 and leads to succinate-dependent stabilization of the transcription factor HIF1α, which masters several pro-neoplastic programs (65, 69). Downregulation of cytochrome oxidase activity relies upon the inhibitory interaction between TRAP1 and Src (66). **(B)** TRAP1 inhibits ROS generation by SDH and cytochrome oxidase (66, 70). This, together with the interaction with other chaperones, such as cyclophilin D (CyP-D) and heat shock protein 90 (HSP90), inhibits opening of the permeability transition pore (PTP), which requires conformational changes of the ATP synthase and leads to cell death (71, 72). Therefore, TRAP1 protects tumor cells from cell death stimuli.

In the last few years, several reports have demonstrated that TRAP1 is involved in the metabolic regulation of tumor cells. The first suggestion came with the observation that mitochondrial HSP90 proteins, including TRAP1, maintain energy homeostasis in transformed cells by inhibiting nutrient-sensing AMP-activated kinase, autophagy, and unfolded protein response of the endoplasmic reticulum (64). These authors proposed that the effect of TRAP1 activity on cell metabolism was that of maintaining proteostasis, i.e., organelle integrity and energy conservation. In particular, under stress conditions TRAP1 would impede ATP depletion and the consequent autophagy induction. A more mechanistic comprehension of TRAP1 metabolic functions has been achieved when two different groups have reported that TRAP1 contributes to the switch toward aerobic glycolysis, i.e., decreased OXPHOS activity together with enhanced glucose utilization. They demonstrated that TRAP1 deficiency increases fatty acid oxidation and accumulation of TCA intermediates in neoplastic cells (65, 66), and that TRAP1 inhibited mitochondrial respiration by two different but not mutually exclusive molecular mechanisms (Figure 3). On the one hand, TRAP1 inhibits OXPHOS *via* downregulation of cytochrome oxidase, the complex IV of the respiratory chain (66). On the other hand, TRAP1 inhibits SDH, a metabolic enzyme that is both the complex II of the respiratory chain and a component of the TCA cycle (65). This observation supports a model whereby TRAP1 has an important oncogenic activity. Indeed, TRAP1-dependent inhibition of SDH leads to the consequent increase in intracellular succinate levels, which in turn stabilizes the transcription factor HIF1α, as already postulated (67). The transcriptional program mastered by HIF1α stimulates invasiveness of tumor cells together with their angiogenic potential and further amplifies their metabolic rewiring (7, 68), and succinate-dependent HIF1α induction turned out to be essential for the neoplastic growth of several tumor cells (65).

TRAP1 might also contribute to tumor cell survival under conditions of stress normally encountered during neoplastic growth, such as exposure to redox disequilibria or nutrient shortage. In these conditions, TRAP1 both inhibits the permeability transition pore (PTP) (70, 73), a mitochondrial channel whose opening leads to cell death (71), and maintains proteostasis by blocking autophagy, as mentioned (64). In accord with the importance of TRAP1 induction along tumor progression, we have recently observed that its protein levels are markedly increased in very early, pre-neoplastic foci in a model of liver carcinogenesis. In this setting, the enhancement of TRAP1 expression is restricted to lesions that progress to malignancy, where it associates with a marked SDH inhibition that can be reverted by HSP90-family inhibitors (57).

### TRAP1 and Redox Homeostasis: Chaperoning Controversies on ROS in Cancer

The metabolic regulations exerted by TRAP1 could explain its anti-oxidant activity. Mitochondrial respiration is responsible for the generation of a large fraction of cellular ROS (74) and inhibition of respiratory complexes by TRAP1 downregulates ROS levels (66, 70). Thus, TRAP1 relevance in tumorigenesis might also stem from its function of oxidant shield (Figure 3). However, the interplay between redox status and malignancy of cells is a multifaceted one (16, 17). Therefore, the effects of TRAP1 on the neoplastic process could be complex, possibly depending on the tumor type and stage. Indeed, neoplastic cells have to cope with increased steady-state accumulation of ROS and with a quickly changing redox equilibrium, making them vulnerable to oxidative stress. This could rapidly become lethal, for instance, by inducing PTP opening (75).

In tumor cell mitochondria, TRAP1 could be part of a chaperone network involving HSP90, heat shock protein 60 (HSP60), and cyclophilin D (CyP-D) (73, 76), one of the best characterized proteinaceous PTP inducers (72). TRAP1 function in this complex would be to antagonize PTP opening (73). Therefore, these observations were a first clue to decipher the mechanisms by which TRAP1 protects cells from oxidants and contributes to the resistance to cell death stimuli that hallmarks cancer cells (77). It is reasonable to envisage that TRAP1 could inhibit mitochondrial PTP opening independently of a direct interaction with channel components or regulators. In line with this hypothesis, we have recently shown that TRAP1 limits ROS generation by SDH, *aka* the complex II of the respiratory chain, thus increasing the threshold for PTP opening in cancer cells and protecting them from death stimuli (70). Handling of oxidative stress might be extremely important at the beginning of tumorigenesis, to avoid that a deregulated ROS increase becomes detrimental for viability of cells in which adequate antioxidant defenses are not yet fully established. Accordingly, in the hepatocarcinogenesis model in which TRAP1 levels are induced from the very early stages, malignant cells build up an anti-oxidant program mastered by the transcription factor Nrf2, which also affects TRAP1 expression (57). Hence, in this tumor type a strong correlation exists among early TRAP1 induction, Warburg-like metabolic rewiring and protection from oxidative insults, and these adaptations are required for neoplastic progression to malignancy.

However, a ROS surge could favor tumor growth, as it damages nucleic acids and leads to genetic instability, a crucial adaptive strategy for increasing malignancy that could be particularly gainful for advanced neoplasms (78). Therefore, the anti-oxidant effect of TRAP1 could hamper neoplastic progression in specific tumor types or stages. Accordingly, TRAP1 expression levels inversely correlate with tumor grade in cervical carcinoma, clear cell renal cell carcinoma (66), and high-grade ovarian cancer (79).

Changes in redox equilibrium are also involved in the regulation of cell invasiveness. Again, contradictory reports show that the absence of TRAP1 favors *in vitro* cell invasiveness (80) through an increase in ROS levels (66) and that cell motility and invasion of prostate cancer and glioblastoma cells are promoted by TRAP1 in conditions of limited nutrient availability (81). Moreover, the anti-oxidant activity of TRAP1 could contribute to cancer cell resistance to chemotherapeutics that elicit oxidative stress, especially in colorectal and breast cancer models (47, 82, 83). Accordingly, TRAP1 interaction with the Ca^2+^-binding protein Sorcin was reported to enhance both the stability of the chaperone and resistance of tumor cells to antineoplastic compounds (84).

### TRAP1 and Post-Translational Modifications (PTMs): Mitochondrial Signaling In and Signaling Out

Chaperone activity is required to face a variety of cellular stresses, in order to avoid damage by maintaining protein stability and function, thus ensuring cell survival (85). Therefore, many chaperones have many layers of regulation, both at the transcriptional and at the protein level.

Several lines of evidence indicate that TRAP1 is target of several PTMs that could influence its stability and activity under conditions of cellular stress. A first clue to understand this kind of regulatory events came with the demonstration that TRAP1 is a phosphorylation target of PINK1, a Ser/Thr kinase that can associate to mitochondria. PINK1-dependent phosphorylation leads to an increase in TRAP1 anti-oxidant function (86), even if the relevance of this PTM in tumorigenesis remains unknown. TRAP1 is also Tyr-phosphorylated and this is abrogated by the c-Src inhibitor dasatinib, strongly suggesting that TRAP1 is a target of the mitochondrial fraction of c-Src (Figure 3) (66). The interaction between TRAP1 and c-Src would inhibit the kinase, in turn leading to inhibition of the complex IV of the respiratory chain, a c-Src phosphorylation target (87). Interestingly, Tyr kinases of the Src family are activated upon oxidative stress and can integrate mitochondrial redox signaling pathways (88–90). By inhibiting a respiratory complex *via* c-Src inhibition, TRAP1 would keep ROS levels low, whereas knocking-down TRAP1 expression would stimulate ROS-dependent migration and invasion of tumor cells *in vitro*, again suggesting the possibility that the impact of TRAP1 on tumorigenicity might be context-dependent.

These reports open the reasonable perspective that TRAP1 PTMs could be crucial in tuning metabolic adaptations of tumor cells. Further hints were provided by recent observations that link TRAP1 phosphorylations to Ras/ERK signaling, a kinase transduction pathway whose hyperactivation has a pivotal importance in a variety of tumor types (91, 92). TRAP1 phosphorylation by BRAF, the first Ser/Thr kinase activated downstream to Ras, was associated with resistance to apoptosis in colorectal carcinoma models (93). More mechanistic details came with our recent observation that TRAP1 is phosphorylated in an ERK-dependent way on two Ser residues, Ser511 and Ser568 of the human protein (69). We found that cells endowed with deregulated induction of the Ras/ERK1/2 signaling pathway have a metabolic switch toward aerobic glycolysis that is regulated by a fraction of active ERK1/2 located into mitochondria. Mitochondrial ERK1/2 forms a multimeric complex with TRAP1 and SDH in which the chaperone and the kinase display a reciprocal interaction. ERK1/2 phosphorylates TRAP1, thus stimulating TRAP1 inhibition of SDH activity and crucially contributing to cell metabolic rewiring. In turn, TRAP1 stabilizes mitochondrial ERK1/2, maintaining the kinase active even under stress conditions (69). These observations were made on cells where the Ras/ERK1/2 signaling pathway is deregulated following loss of the Ras GTPase-activating protein neurofibromin, encoded by the NF1 gene. Biallelic inactivation at the NF1 locus is one of the most frequent cancer-associated mutations in a wide array of tumor types and characterizes the tumor-predisposing genetic syndrome neurofibromatosis type 1 (94). Abrogation of TRAP1 expression completely ablates tumorigenicity in cells lacking neurofibromin (69). Therefore, we propose that hyperactivation of a Ras/ERK/TRAP1 signaling axis might be an oncogenic determinant in a large spectrum of neoplastic cells by switching their metabolism toward an aerobic glycolysis phenotype.

Reversible cysteine S-nitrosylation is a redox-based PTM consequent to nitric oxide bioreactivity (95). TRAP1 cysteine 501 residue is S-nitrosylated in HCC cells, as highlighted in cells lacking S-nitrosoglutathione reductase (GSNOR). GSNOR regulates the levels of proteins undergoing S-nitrosylation by catalyzing S-nitrosoglutathione reduction (96), and its deficiency has been observed in certain models of hepatocarcinogenesis (97). S-nitrosylation of TRAP1 primes it for proteasomal degradation. As a consequence, SDH levels and activity rise in HCC cells (98), further confirming the inhibitory role of TRAP1 on SDH and the complexity of metabolic adaptations occurring in the process of neoplastic progression.

Taken together, these observations indicate that PTMs of TRAP1 can be a way by which deregulated transduction pathways funnel signals to the mitochondrial bioenergetic machinery, finely tuning its activity during tumor progression in accordance with the metabolic needs of cells. Notably, this bioenergetic regulation contributes to retrograde signaling, as SDH inhibition by TRAP1 impacts on the nuclear transcriptional profile of cancer cells through succinate-mediated HIF1α stabilization, thus promoting cancer growth (65). Therefore, TRAP1 might have a central role in shaping inward- and outward-moving mitochondrial signals in cancer cells and in setting the metabolic adaptations they need to thrive.

## Targeting TRAP1 as a Strategy for Cancer Treatment

**Figure 4:**
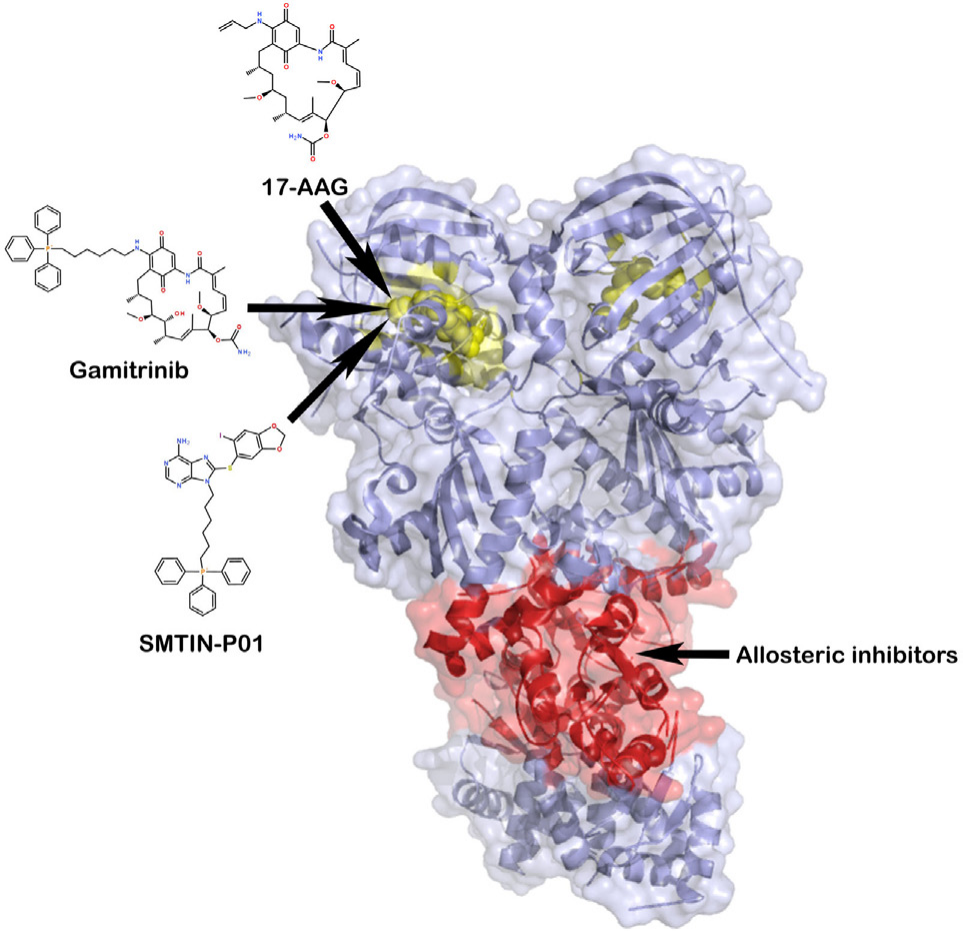
**TRAP1 inhibition by small molecules**. TRAP1 inhibitors can act either at the level of the ATP-binding pocket (the yellow portion of the two protomers), with the aim of blocking ATP hydrolysis, or at an allosteric druggable site, in analogy with heat shock protein 90 (101, 102). In red, it is represented a potential target of allosteric inhibitors at the middle domain:C-terminal domain interface.

Considering the above observations, it is reasonable to envisage that TRAP1 is a good target to develop novel therapeutic approaches for cancer treatment. So far, the strategy used to inhibit its chaperone activity has been to exploit molecules already used as HSP90 blockers. These compounds inhibit ATP binding at the HSP90 N-terminal domain (Figure 4) (99, 100) and are active on TRAP1 too, given the high similarity of its ATP-binding pocket with that of HSP90. The approach developed to increase the selectivity of these compounds for TRAP1 has been to link them to moieties that facilitate permeability across mitochondrial membranes and accumulation in the organelle.

### Targeting HSP90-Family Chaperones in Mitochondria: Moving Toward Organelle-Specific Inhibitors

The first designed molecule that has shown ability to enter mitochondria and target the mitochondrial pool of HSP90s has been shepherdin, a synthetic peptidomimetic inhibitor of HSP90 ATPase activity (103). To accumulate inside mitochondria, shepherdin utilizes a highly positive charged *Antennapedia* cell-penetrating moiety fused at its amino terminus (104). Shepherdin prompts apoptosis probably *via* PTP opening, as it induces cell death in a CyP-D-dependent manner, causing organelle swelling and depolarization and release of cytochrome *c* into the cytosol. Shepherdin has shown efficacy against tumor cells both *in vitro* and in xenograft models, but it exhibits some serious limitations for drug development, such as a low half-life due to degradation in the plasma by esterases and capability to induce an immune response (105, 106). It also modulates the expression of a number of cytosolic HSP90 client proteins (103), suggesting that its activity is not restricted to mitochondria and could have unpredictable off-target effects in non-transformed cells. To circumvent these limitations, another class of mitochondrial HSP90 inhibitors has been designed by attaching either triphenylphosphonium (TPP) or 1–4 units of cyclic guanidinium to the HSP90 blocker geldanamycin, to make it organelle-permeable (101). At high concentration these compounds, dubbed gamitrinib-TPP and gamitrinib-G_1–4_, induce an irreversible collapse of mitochondrial functions, cause PTP opening and elicit death in cancer cell lines, while affecting growth in xenograft tumor models (101, 105, 106). Nonetheless, some critical issues must be resolved, such as compound formulation (like their parent molecules, gamitrinibs are water insoluble), dosing, tolerability, and potential side effects on tissues characterized by high levels of mitochondrial respiration. Recently, another putative TRAP1 inhibitor, named SMTIN-P01, was obtained by replacing the isopropyl amine of the HSP90 inhibitor PU-H71 with the mitochondria-permeant moiety TPP. SMTIN-P01 induces mitochondrial membrane depolarization and shows a slightly improved cytotoxicity over gamitrinibs in some tumor cell lines, but it has not been tested yet in xenograft models (43).

In general, all the experiments performed with these compounds must be considered with caution. First, they are based on the assumption that conventional HSP90 inhibitors such as the geldanamycin derivative 17-AAG are excluded from mitochondria, but whether 17-AAG can penetrate into mitochondria is still under debate (43, 65, 73). In addition, several questions about the specificity of these compounds and the mechanism of their antitumor activity have not been answered yet. In this context, it is still unclear whether it is necessary to inhibit single or multiple HSP90 chaperones in mitochondria to obtain clinical relevant anticancer activity. As there is a significant diversity in the ATP-binding affinity between TRAP1 and HSP90, the same compound may not be able to target multiple HSP90 chaperones with the same efficiency, and the degree of selectivity it displays toward different chaperones is unknown. Possible off-target effects of these molecules also depend on whether they can target only mitochondrial HSP90 family chaperones or also their cytosolic counterparts, an issue that is not fully understood. It is therefore mandatory to design highly selective TRAP1 inhibitors that can be used as potential antineoplastic lead compounds.

### Development of Specific TRAP1 Inhibitors: An Innovative Strategy for Cancer Treatment

To achieve TRAP1-specific inhibition, alternative approaches may be proposed to exploit the structural peculiarities of TRAP1. Allosteric targeting of TRAP1 may represent a viable strategy to identify isoform-specific ligands. Allostery is the prime mechanism by which functional regulation can be achieved *via* the activation of specific conformational states that meet functional requirements. Allosteric drugs might display high selectivity, since proteins of the same family with a high degree of sequence conservation in their active sites can have different sequences and structures at allosteric sites. The atomistic understanding of allosteric mechanisms of protein regulation provides the basis for the development of new drug candidates.

In this context, it is possible to characterize the main traits of nucleotide-regulated internal dynamics of TRAP1 using all atom molecular dynamics simulations, with the aim of identifying the most responsive regions located outside of the ATP-site. Such approach has already proven effective in the design of allosteric anticancer leads against HSP90 (102, 107). Allosteric sites are located in the M-domain and in the CTD of TRAP1 (Figure 4) and their stereoelectronic properties can be used to design a new class of small molecule inhibitors. These new leads can represent novel chemical tools to perturb TRAP1 conformational dynamics. Therefore, they could be exploited to study how TRAP1 conformational changes reverberate on the interaction between chaperone and clients, on regulation of signaling and metabolic pathways in mitochondria and eventually on the role played by TRAP1 in tumorigenesis.

## Concluding Remarks

Additional clues about TRAP1 role in tumors can only derive from a more in-depth characterization of its mode of action and regulation. The complexity of TRAP1 effects on tumor cells could stem from several factors, including TRAP1 regulation by several PTMs and possibly cochaperones, and the possibility that it interacts with other client proteins in cells endowed with continuously changing energy needs under the pressure of diverse environmental factors. In this scenario, the possibility of connecting TRAP1 chaperone activity with oncogenic transduction pathways, such as hyperactive Ras/ERK signaling, opens new avenues to elucidate in which tumor types and stages the metabolic rewiring prompted by TRAP1 is pivotal for neoplastic progression. The identification of highly selective compounds to modulate its chaperone activity will be instrumental to dissect how TRAP1 acts in tumor cell mitochondria and to understand its importance as a molecular target for innovative antineoplastic strategies or for the setup of novel combinatorial therapies.

## Author Contributions

IM, CS-M, GC, and AR contributed to writing the manuscript and drawing the figures.

## Conflict of Interest Statement

The authors declare that the research was conducted in the absence of any commercial or financial relationships that could be construed as a potential conflict of interest.
